# Improving Teacher Education to Adequately Deal with Child Sexual Abuse – an Exploratory Study

**DOI:** 10.1007/s40653-026-00881-8

**Published:** 2026-04-22

**Authors:** Lennart Bayer, Maike Cigelski, Justine Eilfgang, Frieda Mensing, Harriet Sewald, Isabelle von Seeler, Simone Pülschen

**Affiliations:** Europa-Universität Flensburg, Flensburg, Germany

**Keywords:** Teacher education, Child sexual abuse, Interview study, Multi-professional

## Abstract

The prevalence of child sexual abuse (CSA) has remained consistently high for years. While children most often disclose experiences of CSA to trusted teachers than to other non-relative adults in their lives, many teachers report feeling unprepared to handle suspected CSA cases. Schools, too, note their uncertainty about whether to intervene and – when they do – which other professionals to involve at what stages of the process. Against this background, this paper aims to identify key CSA intervention training areas that can enable teachers to handle cases of suspected CSA professionally and competently. In contrast to existing research, which tends to focus exclusively on teachers, this Germany-based study draws on interviews with professionals from a wide range of institutions, including schools (*n* = 17), child protection services (*n* = 15), and law enforcement (*n* = 13). Significantly, all of the interviewees highlighted the need for improved teacher education on CSA intervention, with inter-institutional collaboration and knowledge of how to conduct an intervention being the most frequent topics of recollection among those interviewed.

## Introduction

Child sexual abuse (CSA) is a pervasive problem, affecting children of all backgrounds, ages, and genders (Singh et al., 2014; Stoltenborgh et al., 2011). The estimated prevalence of CSA is approximately 11.8% worldwide (Stoltenborgh et al., 2011). CSA is a risk factor for posttraumatic stress disorder (PTSD) (Boumpa et al., 2022; Kendall-Tackett et al., 1993), depression and anxiety (Amado et al., 2015). To help children and young people who have experienced an assault and protect them from further harm, it is essential that they disclose their CSA experiences. Additionally, a timely intervention and social support can reduce negative effects for CSA survivors (van Toledo & Seymour, 2013).

In Germany, where children must attend school until age 15, teachers’ frequent direct contact with pupils often makes them key facilitators in cases of CSA disclosure. Teachers’ close proximity to pupils means they can keep track of individual pupils, any behavioral changes, and develop trustworthy relationships with them that can ultimately facilitate and encourage disclosure (Blakey et al., 2019). Beyond that, teachers are in a position to establish supportive structures for CSA survivors in their classes and to enable each of their students to take part in class to the best of their abilities (Hobbs et al., 2016). Hence, it is especially important that teachers remain aware that CSA might be happening to their pupils and are prepared to respond to it in order to minimize negative effects of CSA.

While becoming aware of CSA is the first and most important step towards being able to provide help, teachers must also know how to react to a disclosure. Research shows that teachers who learn of a pupil’s CSA often feel unprepared for first conversations (Christmann et al., 2017) and feel unsure about how to handle the situation thereafter (Baginsky & Macpherson, 2005; Efrati & Gewirtz-Meydan, 2022). This is not surprising, given longstanding complaints that teacher training has underprioritized CSA topics since the 1990 s (Baginsky & Hodgkinson, 1999) and has been inadequate to date (Al-Zboon & Ahmad, 2016; Blakey et al., 2019). Reacting to CSA always includes uncertainty as a suspicion of CSA is seldom accompanied by clear indicators for CSA, therefore it is necessary to train teachers how to deal with this uncertainty in a professional way. Indeed, recent studies show that CSA-related curricular content of teacher education programs in this area has scarcely changed since the 90’s, highlighting a need to develop various CSA topics in teacher training curricula. This study aims to identify potential training content areas that can enable teachers to confidently and effectively respond to cases of suspected child sexual abuse.

## Current Research on Dealing with Suspected CSA in Schools

As noted above, teachers can become trusted confidants of children who are experiencing CSA (Blakey et al., 2019; Christmann, 2021) – in fact, a teacher may be the child’s most important non-relative adult confidant in such cases, as a retrospective study by Wager (2015) and a representative survey in Germany by Erkens et al. (2021) suggest. To fulfil this role, teachers need specialized training that enables them to view themselves as possible recipients of disclosure (Draugedalen & Osler, 2022) and also instills their confidence in this role. Christmann et al. (2017) also assume that teachers who know that their actions can help protecting children are more likely to react confidently to cases of CSA.

Nevertheless, teachers’ own reactions to CSA cases and CSA disclosure vary (Urban, 2019). Whilst some teachers see themselves as responsible for intervening in such cases, others defer to more experienced professionals (Sigad & Tener, 2022; Urban, 2019). Many teachers, including freshly certified ones, report a lack of training in child protection and specifically in CSA prevention and intervention (Al-Zboon & Ahmad, 2016; Blakey et al., 2019; Kenny, 2001). Even more troubling, Glammeier (2019) shows that teachers who buy into CSA myths often have adverse reactions to a disclosure, and tend not to believe children when they disclose their experiences of CSA. Similarly, Márquez-Flores et al. (2016) find that many teachers are convinced of the frequent falsity of CSA accusations, with some even attaching a specific profile to CSA perpetrators.

In researching ways to address these issues, studies show that targeted workshops and training modules can effectively boost teachers’ knowledge of child sexual abuse, their confidence to act, and their willingness to report cases of CSA cases to the police (Baginsky & Macpherson, 2005; Hazzard, 1984; Madrid et al., 2020; Mathews, 2011). Such research suggests that boosting teachers’ confidence in their own ability to react to suspected abuse may be as necessary for CSA as it is for cases of physical or emotional abuse, for which such training is considered essential (Yanowitz et al., 2003).

Although teachers generally express positive attitudes towards CSA education (Márquez-Flores et al., 2016), there are structural barriers that impede their ability to react appropriately. Draugedalen et al. (2021) note that lack of time presents a significant challenge, preventing teachers from adequately fulfilling their child protection responsibilities. To address this, educational policies must support teachers who seek to educate themselves about handling suspected CSA by ensuring that sufficient time is allocated for these important tasks. Encouraging school staff to collaborate with professionals from child protection or law enforcement whilst also establishing multi-professional teams at the onset of a CSA intervention can also benefit schools (Christmann et al., 2017; Draugedalen et al., 2021). In such situations, teachers can receive expertise in topics for which they may feel underprepared (Christmann et al., 2017). One recent study has also found that collaborating with child protection and law enforcement professionals can prompt school professionals to take more decisive action in CSA cases (Draugedalen et al., 2021).

In identifying possible topic areas for future teacher education, Glouchkow et al. (2023) suggest that teacher education modules address differences in perceiving various types of maltreatment and police reporting procedures as two key training areas. Other studies suggest including general information on the subject, guidance on how to approach children who may be affected by sexual abuse, and information on how to collaborate with child protective services and the criminal justice system (Baginsky & Macpherson, 2005; Márquez-Flores et al., 2016). In any case, it is important to recognize the institutional potential of schools to increase the visibility of child sexual abuse (Admon Livny & Katz, 2018). In this regard, Germany’s Standing Conference of the Ministers of Education and Cultural Affairs (KMK) stresses the importance of schools as places of expertise on CSA (KMK, 2023). Training teachers in CSA is one way to potential this institutional role.

### Legal Framework of CSA Intervention in German Schools

In Germany, there are no laws mandating the reporting of suspected cases of CSA. The responsibility for penalizing CSA falls under the jurisdiction of two distinct institutions:

1. The youth welfare office (YWO) is predicated on family law. It has a protective mandate under § 8a SGB VIII, which requires it to assess and manage child welfare risks when it becomes aware of significant indications of harm.
2. Police and prosecutors handle criminal investigations and prosecutions under the German Criminal Code in cases of CSA (StGB §§ 176-176e) among other offences against sexual self-determination (Division 13 StGB: Offences against sexual self-determination).

§ 8a SGB VIII also establishes the legal foundation for the issuance of a protective mandate for children and adolescents within the confines of schools and other public educational institutions. Recently, the focus on child protection in schools has increased with the introduction of the German federal Law on Cooperation and Information in Child Protection (KKG) which allocates a greater share of responsibility for the safeguarding of children and adolescent to professionals within the school environment (§ 4 KKG). Furthermore, certain German states – including Schleswig-Holstein (§ 4 (11) SchulG), the focus of this study – have implemented jurisdiction for compulsory development of a child protection plan that includes a plan to action in cases of suspected CSA. Consequently, educational institutions and their personnel in Germany are confronted with mounting pressure to implement comprehensive child protection strategies.

## Aims and Research Questions

Teachers are important actors in cases of CSA because of their daily contact with their pupils. As noted above, teachers’ self-reported feeling of unpreparedness to handle these situations when they arise is only one of the many reasons uncovered by research to promote teacher education in CSA and its prevention. We recognize that dealing with suspected CSA will cause frequent insecurities for responsible teachers as there are no clear indicators for perceiving CSA. A training curriculum can contribute towards dealing with CSA professionally even though the issue itself poses an inherent insecurity. Thus, there is an urgent need for a training curriculum that can address ways of reacting professionally to suspected CSA for teachers and identify key content areas for training in responding to suspected CSA. This is especially true in Germany, where knowledge of CSA is still largely absent among teachers.

To address this gap and identify further training needs for teachers on the topic of CSA, we conducted exploratory interviews with professionals from schools, child protection, and law enforcement. The focused results of these interviews are presented in this paper. Combining the interview data from this mixed group of professionals yielded complementary data. On one side, the feedback from teachers and school staff provided valuable insights into daily practices in schools. The group of teachers in our study consists of both teachers (*n* = 5) and principals (*n* = 5) who usually carry a teaching obligation in addition to their administrative tasks in the German school system and were qualified teachers. For their part, professionals from other sectors who were experienced in handling cases of CSA contributed vital expertise on key requisites needed by teachers in this area to effectively handle cases of CSA. The interviews aimed to answer the following research questions:

1. What uncertainties do teachers perceive in themselves regarding their handling of CSA cases, and which topics do they consider important to teacher education on CSA?
2. What uncertainties do non-school professionals perceive when it comes to intervening in suspected cases of CSA?
3. What educational and professional requirements for teachers do they identify as required topics for teacher education in this area?

To address our research questions and to analyze the data we used a definition of CSA that closely aligns with the definition of CSA in the German Criminal Code (StGB §§ 176-176e). This definition of CSA includes both contact and non-contact abuse as well as grooming behavior initiated by adults. We did not specifically ask for knowledge about cases of sexual violence among peers in the interviews. Nevertheless, some interviewees mentioned assaults among peers, showing that this is a prevalent topic among professionals relevant to child protection.

## Method

We addressed these questions, by conducting semi-structured expert interviews (*n* = 50) within the framework of an overarching research project. More articles are based on the same sample but discuss different topics in the context of CSA (see Bayer et al., 2025a, b; Eilfgang et al., 2024). To address the questions as comprehensively as possible, we interviewed two groups with complementary areas of expertise: (1) school personnel (i.e., experts in schools as institutions), and (2) non-school professionals (i.e., experts in CSA handling suspected CSA and intervention). To gather insights into the professionals’ knowledge of CSA (Bogner et al., 2014; Misoch, 2019), we chose a semi-structured interview format. Data was collected from November 10th 2021 until May 1 st 2022. Interviewees included school personnel (*n* = 17, teachers, principals, school social workers, school psychologists), professionals from child protection (*n* = 20, “insoweit erfahrene Fachkraft” [referred to as “child protection experts” in Germany], youth welfare offices, counselling centers, and psychosocial court support services), and law enforcement (n = 13, police officers, prosecutors, attorneys). Hennink and Kaiser (2022) report that conducting 9–17 interviews in a homogenous sample usually leads to saturation in regards to answering qualitative research questions. We reached this threshold for each professional subgroup relevant to our research questions. The interview protocol, based on that of Kruse (2015), structured the interviews in several thematic parts to ensure consistent coverage of topic areas across all interviews. The interview protocol addressed three key topics among others:

1. **Experiences with CSA cases** (“Have you personally encountered a case of child sexual abuse?”).
2. **Attitudes towards dealing with CSA cases** (“A child has confided in you, and now this has raised a suspicion of child sexual abuse. How do you proceed?”).
3. **Further training needs of teachers and other school professionals** (“What qualifications do you think teachers need to have so that they can handle suspected cases of child sexual abuse adequately?”).

For more in-depth information about the interview protocol and other questions asked about these three topics, view the appendix. The complete interview protocol has been published in another journal article [blacked for peer review]. Other topics, like mental health and knowledge of law enforcement, were also covered in the interviews; however, as the information from these interview segments was of limited relevance to this paper, it will be covered in future articles.

Prior to the interviews, the interview protocol underwent rigorous two-phased pilot testing in real-life conditions (Kruse, 2015). Real-life conditions meant interviewing professionals from school, child protection and law enforcement using a preliminary version of the interview protocol to ensure that the protocol works as intended in the target population (Kruse, 2015)^1^. Four different interviewers conducted the interviews, predominantly face-to-face but also (exceptionally) via videoconferencing using Cisco Webex. All participants received detailed information about the project and gave their informed consent prior to each interview. The interviews were transcribed by research assistants who had been briefed on the rules for transcription. The transcripts were then checked for anonymization and spelling and imported in MAXQDA before socio-demographic data was added as document variables (Kuckartz & Rädiker, 2020).

The interview analysis employed Kuckartz’s (2018) method of qualitative content analysis (QCA), chosen for its usefulness in determining prevalent interview themes and its ability to structure findings in ways that can help inform teacher education. Kuckartz’s methodological framework informed the systematic and iterative development of the coding system. In the first phase of analysis, the research team developed codes deductively, based on the topics of the interview protocol. Subsequently, the team developed additional codes inductively, drawing directly from the observed content while coding eleven interview transcripts. When the first version of the code system was finished, the second phase of analysis started. Working in pairs, eleven more interviews were coded consensually using the first version of the code system (Kuckartz, 2018). After the consensual coding was finished, the code system was used to code the remaining 28 interviews. The final code system is shown in Table 1.

**Table 1:** Code system

Main code	Subcode	Definition	Intercoder agreement
Collaboration*	-	All sections that focus on needs for teacher education regarding collaboration with internal as well as external partners. This code also includes sections where school professionals show missing knowledge regarding collaboration.	96.36
Intervention	-	The category comprises needs for teacher education regarding intervening when suspecting CSA.	-
	Dealing with suspected CSA	All sections that focus on the dealing with a suspicion of CSA as necessary content for teacher education as well as sections that focus on the potential role of teachers and other school professionals in this process CSA.	96.15
	Talking to children*	All sections that focus on talking to children as a necessary content of teacher education. It also includes sections in which teachers or other school professionals express missing knowledge on talking to children in cases of CSA or no awareness for the problem of suggestibility.	88.89
	Procedure	All sections that focus of the procedure of an intervention in CSA cases as necessary content of teacher education.	77.78
	Legal requirements*	All sections that focus on legal requirements as a necessary content for teacher education. It also includes section in which school professionals utter missing knowledge on legal proceedings surrounding CSA cases.	81.48
	Dealing with affected children	All sections that focus on the need for teacher education to include dealing with affected children as well as sections in which teachers express ideas for dealing with (possibly) affected children and ideas for supportive measures in school.	82.35
	Dealing with potential perpetrators	All sections that focus on dealing with potential perpetrators as a content of teacher education. It also includes sections, in which teachers or other school personnel express missing knowledge on dealing with potential perpetrators.	92.31
	Working with parents*	All sections that focus on working with parents in CSA cases as a necessary content of teacher education.	90.91
		**Total intercoder agreement**:	89.64

Codes marked with an asterisk (*) were derived deductively based on the interview protocol. All other codes were developed inductively from the material

To determine code system quality, the team used MAXQDA to calculate the intercoder agreement following the method outlined by Kuckartz (2018). This yielded an overall intercoder agreement of 89.64%, with a Cohen’s Kappa coefficient of 0.88. This high level of agreement and kappa value indicate the very good reliability of the final code system used for QCA (Kuckartz & Rädiker, 2022). The intercoder agreement for each individual code is shown in Table 1.

## Sample

Socio-demographic data for the interviewees was collected through a questionnaire completed by each participant prior to the interview. This self-reported socio-demographic data can be seen in Table 2.

**Table 2:** Socio-demographic data

School (*n* = 17)	Gender	Age (Ø)	Experience with CSA cases
5 teachers (Te)	3 male 2 female	44.8 years	5 inexperienced^2^
5 principals (Pp)	2 male 3 female	50.75 years	3 inexperienced 2 experienced
5 school social workers (SSW)	1 male 4 female	50.4 years	1 inexperienced 4 experienced
2 school psychologists (SP)	2 female	39.5 years	2 experienced
Child protection (*n* = 20)			
5 child protection expert in Germany (CPE)	1 male 4 female	47.8 years	5 experienced
5 youth welfare office (YWO)	1 male 4 female	45.4 years	5 experienced
5 counselling centre (CC)	2 male 3 female	45.4 years	5 experienced
5 psychosocial court support (PCS)	5 female	47.6 years	5 experienced
Law enforcement (*n* = 13)			
5 police officers (Po)	3 male 2 female	45 years	5 experienced
5 prosecutors (Pr)	1 male 4 female	48.6 years	5 experienced
2 attorneys (At)	1 male 2 female	51.3 years	3 experienced

^2^One teacher has experience with handling teen dating violence

The interviewees from school worked in primary, secondary and special education settings. To ensure the participants’ anonymity, we chose not to report their institutional affiliations in detail. Much of the CSA-specific knowledge accessed during the interviews is unrelated to the age of children and adolescents (i.e. collaboration, procedure, working with parents).

## Results

In the following section, which organizes the results of our content analysis, we categorized our results by code (referring to the codes discussed earlier). Our findings for school professionals are presented first, followed by those for child protection and law enforcement professionals for each code. The interviews are cited in the following format: professional abbreviation, unique person (interviewee) number, number of the segment being referenced.

## Collaboration

All but two interview partners (At03, Pr05) cite “collaboration in CSA cases” as an important content area for teacher education. Both school and non-school professionals stress the need for teachers to know and keep in touch with local experts as collaboration partners in CSA cases. One teacher stressed the relief they would feel from knowing how and with whom to collaborate in cases of suspected or disclosed of CSA, when teachers are obliged to react, and mentioned the topic’s strong relevance to teacher education:

I think it would be a relief to know how to act in such a case [of suspected CSA], and which institutions you have to involve. To know which places to contact, if interdisciplinary collaboration is necessary. I think this is very, very important. (translated quote; Te01/132)

All teachers expressed a desire for additional education about how to identify potential partners in CSA cases^1^, or made statements that revealed their confusion about local collaboration partners in CSA cases, indicating a need for further instruction on the topic^2^. As one teacher noted:

I know there is the youth welfare office and I don’t know, maybe I would ask the police who to ask [in a CSA case]. They will certainly tell me ‘Call child protective services.’ I’d say‚ I’m a teacher from this school and this happened. Who can I call?’ (translated quote; Te04/38).

Some school professionals also point to the need for a clearer understanding of the respective roles and responsibilities of collaboration partners^3^. Teachers should also know about school-internal collaboration partners, they stressed; school social workers, for example, were deemed to play a very important role in handling suspected CSA^4^. However, only a limited number of school professionals mentioned the child protection experts who advise other professionals via anonymously presented case descriptions. These experts, were perceived as offering a low-level support in CSA cases^5^. Interestingly, no teachers identified child protection experts as potential collaborators.

Now, the results regarding the non-school professionals will be presented. As mentioned before, they also stressed the importance of collaboration in cases of (suspected) CSA. In the words of one professional from the youth welfare office:

Fundamentally, [teachers] should know about our capabilities. Boundaries and capabilities of our actions. That is, I think, a reoccurring topic, no matter the area, but sometimes our capabilities are getting overestimated. And especially then, there is a corresponding weight of expectation and if that is not fulfilled, it sometimes leads to frustration and resignation. (translated quote; YWO01/72)

For their part, non-school professionals stressed that teachers should know the capabilities and limits of their collaboration partners in CSA cases^6^. One police officer highlighted the different tasks of child protection institutions versus law enforcement professionals:

Yes, I think the youth welfare office is the right contact, because in contrast to us police officers, they don’t have to comply to the principle of legality of a report. I think it is important that every abuse gets reported, but still the youth welfare office has another view, namely on child’s welfare. ‘What is the best option for the child here and now?’ [.] That is of course important and therefore I think it is right when a teacher who cannot accomplish an intervention in a CSA case, who is not a detective and doesn’t have the knowledge like professionals from the youth welfare office, contacts a fitting institution. And I think the youth welfare office is the right institution, which in turn need to know what other institutions do during a CSA intervention. (translated quote; Po01/33)

Child protection professionals seconded the opinion, voiced by school personnel, that school social workers are very important contacts for teachers in CSA cases^7^. In contrast to school staff and especially teachers, child protection workers highlighted the special role that child protection experts in Germany are given when it comes to collaborating in CSA cases, naming them as very important collaborative partners for teachers^8^.

Given that nearly all interviewees cited collaboration as a critical subject area for teacher education, and the diverging statements between school and non-school professionals, the existence of an urgent need to include this topic in CSA training curricula seems clear.

### Intervention

The main code on “Intervention” comprises all interview segments focused on the need to further qualify teachers on intervention-related issues in cases of suspected CSA. This main code was divided into seven subcodes, each of which will be analyzed individually.

### Dealing with Suspected CSA

As noted above, the lack of specific signs for recognizing CSA makes handling such cases challenging and requires careful handling. In discussing this, one school psychologist underscored the school’s important role in CSA intervention:

Well, school is - I think at least - an institution, where things may attract attention for the first time, where children might disclose their experiences or say something. Their responsibility is to stay aware and observe the children, if anything unusual stands out. For CSA, as with all other topics, there should be a lot of attention paid to it, which means that teachers should be trained to look more closely at certain points and also to know how to address things in case of doubt. (translated quote; SP01/32)

For teachers and other school professionals to effectively handle suspected CSA, participants stressed, they must be trained to know how to proceed in such a case^9^. Teachers themselves voiced their desire to know how to deal with suspected CSA if they detect signs of stress, or suspect CSA, in relation to a specific pupil. In the absence of such training, a teacher might take recourse to pre-existing ideas from their own life experience:

I think, I have certain things on my mind that could happen if I experienced CSA, and I would maybe ask about these things. ‘Do you meet up with other people? Do you like being with other people?’ or: ‘How are you sleeping currently?’ (translated quote; Te05/48).

Instead of relying on presuppositions about certain behavioral changes, teachers should be taught that CSA is almost never very clearly recognizable and often relies on frequent, unbiased communication with children^10^.

In contrast, non-school professionals demonstrated awareness of the difficulties in handling suspected CSA:

Well, school is often an institution where children disclose their experiences of child sexual abuse for the first time or where there is a first suspicion. When I’m thinking about cases of consulting, they often appear in schools. And then it would be right that teachers know ‘Okay that is a warning signal.’ Even though there are no clear warning signals, they should know what to look for. (translated quote; PCS01/56)

Other professionals stress the importance of school in handling suspected CSA^11^. They also underscored the need for teachers to be taught in this regard:

[…] But I think that teachers need to be shown during their studies what consequences there are for victimized children. Potentially, what changes in behavior can be prevalent when children have been victimized, to know what to look for. If children don’t initiate a disclosure by themselves, but instead teachers need to be sensitive. Which signs should especially sensitize teachers. I think that’s what needs to happen first of all: Provide information and sensitize teachers. (translated quote; Po05/58)

Non-school professionals also stress the need for teacher awareness of sudden changes in their pupils’ behavior, as a way to enhance their CSA preparedness^12^. However, they warn that there are no clear indicators for CSA, emphasizing that teacher education should specifically address this fact^13^. Thus, teachers need to know how to respond when a child discloses their experiences^14^. On the topic of disclosure, one police officer stressed that teacher’s approachability to their pupils is just as important as their knowledge of how to deal with a suspected case of CSA:

I think that it is important, apart from the perception, I think that it also depends a bit on the personality of the teacher, which perception he has. I think we all know people who somehow attract people to approach them with problems and others somehow have such a wall around them that no one comes forward. (translated quote; Po01/31)

### Talking to children

Teachers expressed a lack of confidence for initiating first conversations with potentially affected pupils:

[…] I think, even though I have always dealt with the topic, but I don’t know what the current state is, what would really be advisable. […] A first conversation, no I wouldn’t dare to do that. I would, if I wanted to conduct a first conversation, like to take a course on it. I would like to get advice beforehand. (translated quote; Te02/45)

Other teachers echoed this sentiment, reporting their feeling of unpreparedness to conduct a first conversation in such circumstances^15^. They also expressed a desire for course training on how to talk to children in such cases^16^.

Teachers, it would seem, want but are not getting the kind of training that would prepare them to talk to children effectively. This need was also voiced by other school professionals^17^, some of whom appeared unaware of the dangers of suggestively influencing children^18^.

For their part, professionals from child protection and law enforcement also advocated for more teacher education on the topic^19^.

Well, fact is, you have to know some types of questioning and how to use questions. You need to know what it means to be suggestive and how not to be suggestive. You need to know what open-ended questions are. You need to know what certain questions trigger in an interviewee. (translated quote; At03/21)

Building on these findings, our study emphasizes the importance of providing teachers with basic information about avoiding suggestive questioning and influencing children with one’s presuppositions as opposed to the desired open-ended approach to CSA^20^. Teacher education training has to cover the topic of suggestibility, they stressed, as a key challenge during (first) conversations with potentially affected children and youth^21^. In discussing the importance of talking to children, one interviewee mentioned potential to cause further harm as important reason to train teachers in this subject area:

Probably, it would be important in this context that they [teachers] actually take part in further education where they learn how to talk to children and teenagers in these specific situations without causing further damage. Because that can happen. (YWO02/22)

These comments underline how challenging it can be to talk to children in cases of suspected CSA and the potential for negative consequences (e.g., retraumatization) that can result from poor interviewing techniques. Teachers’ demand for training that prepares teachers for these special conversations was echoed by their external partners from child protection and law enforcement, who expressed hope that increasing teacher education would positively impact on the way they talk to children who may have been affected by sexual abuse^22^.

### Procedure

Some of the teachers, aware of their lack of knowledge on intervention procedure, took this as a reason to demand its inclusion in CSA training^23^. The request for a ‘how-to’ guide was very prevalent in interviews with the school professionals:

I think, [I would wish for] an actual guide to action. I think, us teachers, we really like having a guide to action or a guideline. I would like to know exactly, when to do what? And whom to ask? What are possible places to contact and so on. It became clear, that we currently don’t have this and we are working on it right now. (translated quote; Te05/78)

Other teachers and their principals mentioned the need for training to include a ‘how-to guide’ for teachers^24^. A school social worker shared an experience where teachers would rather push away the responsibility for acting upon a CSA case instead of doing something themselves after a pupil at their school disclosed their experience of CSA:

[The interviewee described the disclosure of a student during a poetry slam in a previous part of the interview.] As I mentioned, this story with the poetry slam for example, there were colleagues who came and saw me and said to me: ‘Well, now you have something to do next week.’ And of course the principal contacted me: ‘Are you going to act on that?’ […] They perceive that and talk to me, also with hindsight something like: ‘Have you been there now?’. And then I tell them as much as I am allowed to tell. (translated quote; SSW01/68)

The interviews also revealed a strong feeling among child protection and law enforcement officials that teachers lack guidance. They, too, frequently requested a practical guide for teachers on how to respond to CSA cases^25^:

First of all, the question is whether there is a practical guide […] Actually, if the school is well prepared, there should be a clear, transparent guide to action which is used when it comes to resolving a suspicion. (translated quote; CC03/4)

The interviewees criticized teacher preparation in the area of CSA intervention and stressed the need to include the topic in teacher education^26^. As one police officer put it:

[…] I think you should focus on the process that takes place after a suspicion of CSA appeared and to give teachers a sense of security in their own actions. Teachers should know, about their responsibilities […]. I would think a concept would be ideal, where there is a concept in the drawer, that gets pulled out and there are steps 1), 2), 3) ‘What do I do? Who is my contact?’ […] This leads to security in your own actions and teachers are able to behave differently when they feel that security. (translated quote; Po01/31)

More specifically, this police officer stressed the need for teacher education to include information about the teacher’s role in CSA intervention processes. A child protection expert, who often does the first consultation for teachers, remarked:

Then we often go into clarifying the roles. Often for teachers we have to clarify exactly what their task is [within the intervention]. (translated quote; CPE01/12)

Some professionals mentioned their experience with teachers who tried to deflect their responsibility for CSA cases onto school social workers, as mentioned above^27^. Non-school professionals also suggested that teachers seek external advice prior to initiating an intervention ^28^.

### Legal Requirements

*Contextual note: Teachers in Germany are not required to report CSA cases to law enforcement authorities.*

Some teachers cited legal requirements an important need to further their education on the subject of CSA^29^. The interviewed teachers appeared unsure of their obligation to treat CSA cases with confidentiality:

I don’t know if I have a confidentiality obligation in such a case [of CSA]. (translated quote; Te04/48)

This lack of certainty regarding their obligation to maintain confidentiality also became apparent in other statements by teachers^30^. A general uncertainty about the legal requirements surrounding CSA cases in schools was also mentioned by a principal^31^. Legal responsibilities in cases of CSA also seemed to be a source of insecurity among teachers during intervention. This lack of knowledge seemed to fuel a desire for further education regarding their legal responsibilities during an intervention^32^:

I think it would be good to know when culpability starts. When is school obliged to act. Especially when it comes to digital cases. That’s a topic where all of us are still learning. What is forbidden and what do we have to accept? […] Apart from that, how to act in a network. What are the procedures. This is crucial in my opinion. (translated quote; SP02/94)

Other professionals also mentioned teachers’ lack of certainty about their legal responsibilities in CSA cases^33^. One noted:

[…] But I would wish for them [teachers] to have more professional knowledge regarding legal requirements. You know (questioning), what are rights of the parents? What is the right for data protection? When am I allowed to talk to whom about the child? […] (translated quote; YWO04/84).

Some professionals felt that training on the subject had to cover legal requirements in connection with CSA^34^. Some of these interviewees, who mainly worked in law enforcement, want to include specific content in teacher training about CSA: Information about police work and legal proceedings in CSA cases^35^ as well as data protection^36^.

### Dealing with Affected Children

Dealing with children who have experienced CSA is a critical issue for teachers and other school professionals. As one teacher noted:

I think I would see that the child wasn’t well at that moment, and I would try to act on the surface, but I think I wouldn’t want to reopen any wounds or anything like that. I think I’m not educated for that. (translated quote; Te05/44)

This statement underscores the need for specialized education, as expressed by three of the teachers, affirming their need for training on how to deal with victimized children^37^. However, one teacher and a principal suggested the importance of asking whether the abuse really happened, noting the need to tread carefully when dealing with potentially affected children given (they suggested) that CSA allegations could often be false^38^. Another principal reported that they would not feel comfortable having a first conversation with a potentially affected student^39^. School professionals stressed their own need for more education on the topic.

It also depends very much on the teacher who is responsible for the class. School can be stabilizing for the child, if the teacher is capable of caring for the child and who is able to observe the child. (translated quote; SSW05/72)

Other school professionals stated that children should get support from their teachers and pointed to this as a responsibility for which teachers need to be prepared^40^.

Other non-school professionals also mentioned the importance of teacher support for victimized children and the need for teacher preparedness for this task^41^. Others stressed that teachers are responsible for protecting affected children from further harm^42^. One police officer proposed that teachers should regularly reflect on their behavior towards victimized students^43^.

### Dealing with Potential (adult) Perpetrators

One teacher showed increased uncertainty in dealing with a suspicion that one of their colleagues is suspected to be a CSA perpetrator:

I: (…) What would you do or how would you react if the potential CSA perpetrator was one of your colleagues?

B: (…) Oh (…) Then I would go directly to the principal. I wouldn’t handle that. I wouldn’t want to handle that. I think, that my emotions would be likely to prevent me from being objective, I would certainly give that case away. I wouldn’t think, that I have any expert knowledge to handle such a case in a correct way. (translated quote; Te03/31–32)

More interviewees from school shared this uncertainty^44^. Beyond that, a teacher and a principal stated adverse ways of dealing with a suspicion towards one of their colleagues:

I think I wouldn’t tell my colleague. I would tell the girl or the boy or whatever, I would say: ‘You have to/’ Let’s put it like this: I’ve met people who like to make a scene sometimes. Even though, nothing happened. […] And I really think there are people who do such things, at least for a short time. (translated quote; Te04/40)

If a colleague was a perpetrator. […] I would definitely look for a talk with another colleague, that’s first. Then I would have a look, if it really is urgent, about how to get my [suspected] colleague out of the situation without opening a case. That is quite difficult. That’s even more difficult in the team. I would also get help for that. I wouldn’t really know to be honest. […] I would definitely try to first get my colleague out of this teaching situation. (translated quote; Pp02/18)

Both of these proposed ways of handling suspected CSA run counter to the procedures suggested by seasoned professionals who frequently deal with CSA. That such opinions emerged during the interviews shows the need for further education among school professionals on *dealing with potential (adult) perpetrators*. This need was underscored by one of the teachers interviewed, who requested that how to deal with potential perpetrators should be included in teacher education curricula^45^.

One counsellor stated that they offer training on institutional violence and dealing with perpetrators for schools:

Yes, we offer education on that topic. For example, we also lecture at school development sessions about specialized topics for example. Well, I cover sexual violence in institutions a lot because it is a topic where you are even less likely to be sensitized towards. Regarding perpetrators, where they usually go to. (translated quote; CC02/30)

This statement reveals an institutional demand for training on how to deal with perpetrators, a view even more frequently affirmed by non-school professionals^46^. Moreover, the interviewees proposed that every school should develop its own institutional guidelines on how to deal with potential perpetrators^47^. One counselling center professional expressed a wish for teacher education on the strategies that CSA perpetrators use to get close to children^48^. This enables them to detect strategies like grooming, without having a blanket suspicion^49^.

### Working with Parents

Teachers who encounter suspected CSA of a pupil face the question of whether and when to involve parents in the case, which can prompt feelings of uncertainty:

I think, it’s hard, especially the question who is the perpetrator, the alleged perpetrator? If it was the parents, I definitely wouldn’t notify the parents, at least that’s what my gut tells me, but I honestly don’t know, but then I wouldn’t tell the parents for now. If it was somebody else from their home, siblings, then I wouldn’t directly notify the parents, but instead I would again notify the principal, my colleagues and would get into a conversation about the case. But I think the parents should be involved as soon as possible unless I suspect them to be the perpetrators. (translated quote; Te05/24)

The comment shows a deep doubt about which training is requested by other teachers^50^ and which is also expressed by principals^51^.

One professional from a counselling center noted that teachers also feel unsure of how to deal with parents when discussing a child’s welfare being at risk:

And we say ‘Now something has to happen, we arrange a conversation with the parents.’ And then they don’t know, that they can say something like ‘You now have two weeks to contact an educational counselling center. The deadline. Then we need a response, that something happened.’ Very often, they don’t know that. That they are allowed to set a deadline, that they are allowed to get a release from confidentiality signed by the parents, that they are allowed to inquire and to get anonymous consultation. And then they are just very doubtful. (translated quote; CC02/47)

Experts advise involving cooperation partners in discussions with parents^52^, stressing that such conversations must always take place if the parents are not the perpetrators^53^.

Other professionals stated that CSA interventions should be handled collaboratively between teachers or other school professionals and the parents of the affected child, if they are not perpetrating the abuse^54^. It was also suggested to get support from collaboration partners as to whether and how to involve parents in an intervention^55^.

## Discussion

Drawing on expert interviews, this study has offered exploratory insight into the idea that teachers in Germany should be trained in CSA topics – a wish expressed not only by teachers and school staffs, but also by professionals from child protection and law enforcement who work with CSA cases regularly. Reaffirming the findings of different studies, interviewees expressed a clear need to incorporate CSA and child protection topics into teacher education (Al-Zboon & Ahmad, 2016; Blakey et al., 2019; Kenny, 2001), with each interviewee mentioning at least one aspect of CSA that they felt such training should include. Moreover, some teachers’ statements revealed their knowledge gaps or even adherence to myths and stereotypes surrounding CSA, underscoring the urgent need for further education in this area (i.e. Te02/30/49; Te03/138; Te04/40/68; Te05/48). This is critical information, given that – as noted above – Glammeier (2018) finds that teachers who are misinformed about CSA, or who hold myth-based beliefs about it are less likely to believe children’s disclosures of their CSA experiences. The extent to which teachers rate the disclosing person as trustworthy has also been highlighted for its importance (Glouchkow et al., 2023). In confirming these findings, our results point to the prevalence of myth acceptance and general knowledge about CSA among teachers as important topics for further investigation.

Similarly, our findings for the code for *collaboration* align with previous research (Christmann et al., 2017; Draugedalen et al., 2021; Draugedalen & Osler, 2022), offering a more detailed analysis of the teacher education gap in this specific issue. Interviewee’s statements show the importance of teachers’ knowledge of local collaboration partners, as well as their awareness of the capabilities and boundaries of each organizational role. Our results also offer initial insights into teachers’ most important in-school and external collaboration partners. Whilst teachers and other school personnel school most often mentioned social workers as key partners in this regard, professionals from child protection and law enforcement tended to point to child protection experts. In general, enhanced teacher training would appear needed in the area of *collaboration*.

The interviews also frequently mentioned *dealing with suspected CSA* as a key responsibility of teachers due to their frequent contact with children, which enables them to perceive behavioral changes or stress indicators that could be linked to CSA. This role and its inherent responsibilities align with previous research findings (Blakey et al., 2019; Christmann, 2021; Efrati & Gewirtz-Meydan, 2022; Wager, 2015). For this area, the interview results build on existing research findings by showing that teachers not only need factual knowledge about how to deal with suspected CSA; they also need training on how to facilitate a disclosure addressed to them, and how to increase their approachability for children who want to disclose their experiences. Generally, teachers showed positive attitudes towards receiving and acting on a CSA disclosure. On this topic, non-school professionals stressed the need to educate teachers on the lack of distinct signs indicating CSA victimization and the need to proceed with care when handling a CSA intervention. Our findings also reveal a need for clear training content on next steps after a child has disclosed their victimization. This is part of a broader, critical need to cover the CSA intervention *procedure* when training teachers in CSA, which will be discussed later.

*Talking to children* is very important in cases of CSA intervention, and various studies show an expressed demand for teacher education in this area (Blakey et al., 2019; Christmann et al., 2017; Cowan et al., 2019; Efrati & Gewirtz-Meydan, 2022). Building on these findings, our study stresses the importance of providing teachers with basic information on forensic questioning, the use of open-ended questions, and suggestibility in connection with CSA. The need for training in this area was especially present in statements by law enforcement professionals. For their part, child protection and law enforcement professionals pointed to the dangers posed by poor questioning techniques as the most important reason to include this topic in teacher education.

Previous research studies on CSA teacher training have called for content focused on the intervention *procedure* (Cowan et al., 2019; Scholes et al., 2012), as well as concrete guidelines outlining a teacher’s next steps in cases of suspected abuse (Kenny, 2004). Both of these demands were voiced during the interviews. Several interview sections identified teacher insecurity (Te02/20/22, Te03/76, Te04/26, Te05/78), whilst teachers and many other professionals stressed the need for a practical how-to guide for handling CSA for schools. We found no obvious difference between school and non-school professionals on the importance of training teachers in the CSA intervention *procedure*.

As the *legal requirements* for teachers in CSA cases differ from those of teachers in many other countries, where teachers are required to report suspected CSA to the police, it is harder to compare these results with international research. Still, our interviewees saw CSA reporting procedures as a necessary training sub-topic for teachers, which aligns with international research on the topic (Glouchkow et al., 2023; Madrid et al., 2020). Research findings about teachers’ lack of knowledge of the *legal requirements* related to CSA (Kenny, 2001) was prevalent in this study as well (Te03/40, Te04/48, Te05/40), stressed not only by teachers themselves but also by external partners from child protection and law enforcement. The latter, in particular, stressed the need for teachers to know about their work and about the legal processes that happen in the context of CSA. This gap was not mentioned by school personnel or child protection agents.

Training teachers on *dealing with affected children* was deemed necessary by many of the professionals we interviewed. Teachers expressed this wish, reporting that lack of knowledge on the subject was a source of insecurity for them. Furthermore, the interviews clearly indicated that teachers need training on this topic to be able to recognize the situation and intervene appropriately when they suspect that a child is in need of protection. This is especially important because some professionals from school seem unwavering in their support of myths and stereotypes, which hinders their ability to adequately care for affected children. For their part, teachers reported not knowing what to do when *dealing with affected children.* Teachers and other school professionals need to know about effective strategies to support CSA survivors in schools in order to minimize negative effects of CSA (Hobbs et al., 2016; van Toledo & Seymour, 2013).

For the sub-area *dealing with potential perpetrators*, research shows that intervention is especially challenging for teachers if the potential perpetrator is a colleague (Kenny, 2001). This was also a significant finding in our study, where teachers and other school professionals showed grave uncertainty about how they would handle a situation if the potential perpetrator were a colleague. Interviewees reported their readiness to resort to various, often questionable strategies in this case. In these statements, acceptance of myths regarding the trustworthiness of CSA disclosures was common, confirming interviewee’s own statements stressing the importance of this topic in teacher education.

The topic *working with parents* presented some points of connection with results for *legal requirements*. Teachers, interviewees stressed, need to be taught about when they need to contact and involve parents in case of a CSA intervention. Teachers reported not knowing when and how to involve parents in the intervention, while non-school professionals echoed this wish, emphasizing the need for teachers to understand how to involve parents and when and where to seek external consultation regarding this involvement.

In summary, our findings validate those of previous research on the need for teacher training in CSA, analyzing interview data to offer a more detailed look at key subtopics for inclusion in such training. This nuanced finding was achieved by assessing input from not only from teachers and school staff, but also from non-school professionals who frequently handle cases of CSA.

### Limitations

Our study has several limitations. Since the interviews were conducted in German and later translated into English, information may have been lost during this process. Furthermore, only some selected portions of the interviews were translated into English, potentially altering the original meaning of some segments. This discrepancy presents a challenge for English speakers who seek to align the translated interview excerpts cited in this paper with the full original transcripts when they are published. Despite these shortcomings, it was decided to publish this article in English given the relevance of our results to a broad group of professionals beyond the scope of German speaking countries.

Because the information was obtained through qualitative data, results of this study are not generalizable. They should be seen as preliminary insights that can serve as prompts for further research, drawn from our exploratory approach to CSA and child protection in teacher education. The questions used to elicit statements regarding the role of CSA in teacher education were open-ended and, for the most part, were asked towards the end of the interviews, which may have led some interviewees to leaving out certain details that did not seem as important to them at that moment. Furthermore, interviewee comments on the need for teacher training in the areas of collaboration and intervention must be carefully considered, as this data has yet to be empirically validated. These preliminary findings, which highlight some topics of potential relevance to teacher education on CSA, can be used to develop and validate a core training curriculum in this area.

### Implications for Future Research

As mentioned above, additional quantitative data is needed to support the findings of this interview-based study. Further quantitative research is also needed to confirm the results shown in the interview data. As a follow-up step, one could develop and assess teacher education programs focusing on the CSA topics flagged as important by the interviewees.

In general, future research should examine how schools and school staff handle cases of CSA when a colleague from the institution is the alleged perpetrator. Additionally, it is necessary to investigate the needs of CSA survivors and how to implement a trauma sensitive school environment that meets their needs and allows them to perform academically to the best of their ability.

Training programs for teachers should be developed and evaluated on the basis of these findings. These programs should include information about all topics covered by the interviewees (Collaboration, conversations in suspected cases of CSA, the general procedure of a CSA intervention as well as dealing with specific persons involved in such a case) and a focus on developing an attitude in teachers which enables them to fulfil their role in the network of child protection. As mentioned by many non-school professionals (i.e. Po01/31), it is important for teachers to develop a capacity which enables their efforts in child protection. Currently, such training programs are scarce in German teacher education. Once developed, they should be incorporated in pre- and in-service teacher education programs to promote child protection efforts in schools.

## Conclusion

This exploratory study offers detailed insight into potential sub-areas for inclusion in teacher training on CSA and child protection. As Madrid et al. (2020) have noted, teacher education ideally equips teachers with the knowledge required to effectively handle CSA cases, fostering reflective attitudes towards CSA reporting and giving them a sense of security in their own actions. This makes teacher education on CSA an ideal opportunity to prepare teachers to meet the increasing demands and responsibilities associated with CSA and child protection in general. Furthermore, school professionals are in a position to create a supporting and trauma sensitive environment in schools to reduce the negative impact of CSA.

## Data Availability

The interview data will be made available in German after the project’s funding concludes in July 2026. For earlier access, please reach out to the authors directly.

## Appendix

***A - Relevant questions from the interview protocol***. 

1. **Experiences with CSA cases**

a Have you personally encountered a case of child sexual abuse?

1. **Attitudes towards dealing with CSA cases**

a “A child has confided in you, and now this has raised a suspicion of child sexual abuse. How do you proceed?”

*Alternative for law enforcement:* A child has confided in someone. Now, this has raised a suspicion of sexual abuse and you have been called in. How do you proceed?

*Alternative:* How do you handle a case of child sexual abuse in which a child?

b Let us put aside the previous scenario in which a child has confided in you. Now, imagine that specific observations have led you to suspect that a child may have been sexually abused. However, you have not yet spoken to the child. What do you think needs to be taken into account during this stage of investigating a suspected case?

*Alternative*: Are there specific protocols for what [your institution] must do in such a case? → If yes, what are they, and where are they documented? 

iii. To what extent does [your institution] fight child sexual abuse?
iv. *Alternative:* How important is child sexual abuse in [your institution]?

1. **Further training needs of teachers and other school professionals**

a “What qualifications do you think teachers need to have so that they can handle suspected cases of child sexual abuse adequately?”

*Alternative*: What specific knowledge or skills should be conveyed to teachers to best prepare them for handling suspected cases of child sexual abuse?

**B-References to the interview material**

The following table presents information about which section of the interviews was referenced for statements made in the results section. The number in left column indicates the statement, whereas the column on the right provides information about the: professional group abbreviation, individual interviewee number, number of the specific transcript segment. 

**Table:**

Number	References
1	Te01/132, Te03/100, Te05/78
2	Te02/20, Te04/32/38/82
3	Te03/100, Pp02/58, Pp03/42, SP01/34, SSW02/152
4	Te02/43/79, Te05/96, Pp04/54, Pp05/60, SP01/60, SSW04/74
5	Pp03/60, SSW02/98, SSW04/78, SSW05/76
6	CPE02/50, CPE03/10, YWO01/72, YWO02/112, CC01/54, CC02/6/49, PCS03/28, PCS04/12, Pr01/90, Po01/33, Po03/24, Po04/17, Po05/40/58
7	CPE01/54, CPE02/54/92, CPE04/24, YWO02/66/70, CC01/54, CC02/37, CC05/12, PCS03/26, PCS05/64
8	CPE02/50/54, CPE03/10, CPE05/54, YWO03/60/62, YWO04/86, CC01/18/54, CC02/47/49, CC03/32, CC05/46, PCS03/28, PCS04/12
9	Te01/118, Te02/113, Pp01/78, Pp02/88, Pp04/54, Pp05/88, SSW01/94/114, SSW04/60, SSW05/26/102
10	SP01/34/36, SSW05/26
11	CPE01/50, CPE05/52, YWO01/68, CC04/16, PCS04/12, Pr03/64, Po02/40, Po03/48, Po05/38
12	CPE02/28, CPE05/24/58, YWO01/92, YWO03/62, CC02/71, PCS01/56, PCS03/46, PCS04/12, At02/48, Pr01/114, Pr02/116–118, Pr05/56, Po01/31, Po03/70, Po04/6/27, Po05/58
13	CPE02/26, CPE05/8, YWO03/56, CC02/14, CC04/44, At02/48, Pr03/58
14	YWO05/82, CC01/18
15	Te03/48, Te04/52, Te05/44
16	Te01/118/Te02/113/117, Te03/60/148, Te05/44
17	Pp02/88, Pp05/88, SSW01/114
18	Te02/49, SSW03/20
19	CPE01/22, CPE02/92, CPE03/12/60, CPE05/8, YWO02/22, YWO03/58, YWO05/82, CC05/26, PCS01/56/78, PCS03/46, At01/74, At03/19/21, Pr01/132, Pr03/58, Pr04/64, Po01/16/70, Po02/70, Po04/4
20	CPE01/22, CPE03/16, CPE05/10, At01/74, At02/50, Pr01/132, Po02/70
21	CPE01/22/74, CPE02/30, CPE03/16, CPE05/8, CC01/24, CC04/20, PCS01/24, At01/42/74, At02/50, Pr01/132, Pr02/149/151, Pr05/76, Po02/16, Po03/70
22	CPE01/22; CPE03/16; CC01/24; PCS03/46; PCS04/12; At02/50; At03/21; Pr01/132; Po03/70; Po04/4
23	Te01/118, Te02/22, Te03/144
24	Te01/118, Te02/113/131, Te03/12/76/144, Pp02/58/88, Pp03/74, Pp04/22, Pp05/88
25	CPE04/24, CC03/12, CC05/46, PCS03/30, PCS04/12, Pr01/114, Pr04/64
26	CPE01/12, CPE02/92, CPE03/10, YWO01/92/132, YWO02/112, CC01/18, CC02/14, PCS01/78, PCS04/12, PCS05/80, At02/50, Po01/31, Po03/24, Po04/2, Po05/58
27	CC01/38
28	YWO03/62, CC01/18, PCS05/80
29	Te02/113, Te03/142
30	Te03/40, Te05/40
31	Pp02/38
32	Te02/113, Te03/142, SP02/94, SSW05/102
33	CPE05/56, YWO01/60, YWO03/62, YWO04/84, CC02/37/47, Pr04/32
34	CPE01/12, CPE04/24, YWO03/56, CC01/18/54, CC02/47, Pr03/56
35	PCS01/56, At03/21, Po02/40, Po03/52/70
36	Pr04/32
37	Te01/118, Te02/28, Te05/44
38	Te04/32, Pp02/14
39	Pp05/6
40	Pp04/22, SP01/32/36
41	CPE05/22, YWO03/56, CC01/16, PCS02/60, PCS03/24, Pr05/76
42	CC01/54, CC03/4/8, PCS03/22
43	Po02/42
44	Te02/30/32, Te04/40, Te05/36–38, Pp05/26/28, SSW03/12, SSW04/22
45	Te01/118
46	CC03/4/8/10/12/14, Po02/42/46
47	CC03/8
48	CC02/71
49	CC02/71
50	Te01/118, Te03/30
51	Pp02/88, Pp05/44
52	CPE01/12/52, CPE02/26/92, YWO01/96, CC01/18, CC03/14, CC04/50, PCS03/26, At02/48, Pr01/114, Po04/6
53	SSW05/74, YWO01/92, YWO04/86, Po04/17
54	CPE01/12/52, CPE02/26/92, YWO01/96, CC01/18, CC03/14, CC04/50, PCS03/26, At02/48, Pr01/114, Po04/6
55	SSW05/74, YWO01/92, YWO04/86, Po04/17

## Footnotes

1 The procedure described by Kruse (2015) closely resembles the methodological proposal for interviewing details of experience by Seidman (2006, p. 18).
